# A Comparative Study of Growth Patterns in Microdisks and Normal-Sized Optic Disks Using OCT

**DOI:** 10.1155/joph/2626221

**Published:** 2025-04-20

**Authors:** Rita Gama, Rute Sousa Costa, Tânia Yang

**Affiliations:** ^1^Departamento de Oftalmologia do Hospital da Luz, Lisbon, Portugal; ^2^Consulta de Oftalmologia da Clínica Gama Eye Care, Lisbon, Portugal

**Keywords:** ganglion cell layer, microdisk, optic disk, optical coherence tomography, retinal nerve fiber layer

## Abstract

**Purpose:** To compare the parameters of the optic nerve head (ONH) and inner retinal layer thickness between children and adults with microdisks (MDs) and normal-sized disks (NSDs) using optical coherence tomography (OCT).

**Methods:** A case-control study that included 172 eyes. Four groups of patients were created and matched according to gender and disk size: 41 children with MD (disk size between 1.42 and 1.60 mm^2^), 41 adults with MD, 45 children with NSD (disk size between 1.80 and 2.30 mm^2^), and 45 adults with NSD.

All subjects were imaged with spectral domain OCT. Neuroretinal rim (NRR) area, cup–disk ratio (CDR), cup volume, peripapillary retinal nerve fiber layer (pRNFL) thickness, and macular ganglion cell–inner plexiform layer (mGCIPL) thickness were obtained.

**Results:** There was a statistical difference in the NRR area (*p* < 0.001), average CDR (*p* < 0.001), cup volume (*p* < 0.001), and average pRNFL thickness (*p* < 0.001) between children and adults with NSD. However, comparing children and adults with MD, the only differences found were the NRR area and nasal pRNFL thickness (*p* = 0.009 and 0.010, respectively).

**Conclusions:** In conclusion, the ONH parameters and pRNFL thickness are different in children and adults with NSD. On the contrary, MD belonging to children and adults did not have significant differences in ONH parameters and inner retinal layer thickness.

According to these findings, the evaluation of a glaucomatous lesion in MD should rely on the ONH parameters and an increase in the cup volume must raise the suspicion of glaucomatous damage, because the physiological enlargement of the cup with age in a MD with age is not to be expected.

## 1. Introduction

Microdisk (MD) or small-sized disk is considered when the optic disk has an area smaller than 1.63 mm^2^ measured by optical coherence tomography (OCT). Its prevalence is estimated to be 5%–10%, representing the lower limit of the optic nerve head (ONH) area distribution in a population. These disks are known to have a smaller cup–disk ratio (CDR) and a higher density of nerve fibers known as “crowding of the fibers” that represent a major challenge for the detection of glaucomatous neuroretinal thinning [1, 2].

MD should be distinguished from optic disk hypoplasia, which is a congenital anomaly of the optic disk. Hypoplasia of the optic disk is associated with an absent foveal pit and reduced inner retinal layer thickness at the macula and the optic disk measured by OCT [3]. The main fundus characteristics of optic disk hypoplasia are the peripapillary double-ring sign and the ratio of the horizontal distance between the center of the macula and the disk diameter of 3.0 or larger. In OCT imaging, the hypoplastic disk is smaller than MD, with an ONH area of < 1.42 mm^2^ [4–6].

The ONH area influences most of the parameters measured by OCT. The peripapillary retinal nerve fiber layer (pRNFL) and macular ganglion cell–inner plexiform layer (mGCIPL) thicknesses are positively correlated with the optic disk size, especially in children [7–9]. Also, the ONH parameters are influenced by disk size: children with megalopapilla and normal-sized disks (NSDs) have smaller CDR and a thicker neuroretinal rim (NRR) than adults for the same disk size [10]. How the disk size influences the OCT measurements of MD is still unknown.

The purpose of this study was to compare the stereometric parameters of the ONH and inner retinal layer thicknesses (pRNFL and mGCIPL) belonging to children and adults with MD (Adults MD) using OCT imaging.

## 2. Materials and Methods

### 2.1. Population Study

This case-control study included subjects attending our department of ophthalmology, between February 2013 and July 2015. The study protocol followed the statements of the Declaration of Helsinki, ethics committee approval was obtained (ID 625, CES 08/2024), and written informed consent was signed by all participants before their inclusion in the study. In the case of children under 12 years of age, informed consent was obtained from the parents. Confidentiality of the data was preserved by identifying patients by the medical record number.

A diagnosis of MD was considered when the ONH area in Cirrus® HD-OCT (Cirrus HD-OCT, Carl Zeiss Meditec Inc, Dublin, California) was ≥ 1.42 mm^2^ and ≤ 1.60 mm^2^ and NSDs when it was ≥ 1.80 mm^2^ and ≤ 2.30 mm^2^ [11–13]. If there was a bilateral MD or bilateral NSD, only the right eye was selected for the study. For unilateral MD, only the eye with MD was included. To decrease the impact of refraction errors on the OCT measurements, children and adults were included if the spherical refractive error was between ±1.5*D* and cylindrical ±1.5*D* and the refraction difference between both the eyes was ≤ 2*D* [14].

Four groups of patients were created as follows: Children with MD (Children MD), Adults with MD (Adults MD), Children with NSD (Children NSD), and Adults with NSD (Adults NSD). The children NSD and adults NSD subjects were used as control groups. The patients were matched according to the ONH area and gender.

Optic nerve hypoplasia was excluded based on the following criteria: ONH area < 1.42 mm^2^ measured by HD-OCT, peripapillary double-ring sign, or the ratio of the horizontal distance between the center of the macula and the disk diameter was 3.0 or larger on fundus inspection (with biomicroscopy and OCT) [4–6].

Children and adults were excluded if there were any media opacities precluding imaging technique; congenital optic disk anomalies such as morning glory, optic nerve drusen, or tilted disk; retinal disorders; strabismus or history of occlusion treatment; or there was an inability to undergo the tests. Patients were included if they agreed to participate in the study.

#### 2.1.1. Selection of Children

Children underwent a comprehensive ophthalmologic evaluation that included monocular visual acuity (Snellen chart read at 300 cm), stereoacuity (Titmus fly test), ocular motility examination, cycloplegic refraction with cyclopentolate hydrochloride 1%, biomicroscopy evaluation, intraocular pressure measurement, and fundus inspection. Children were included if younger than 12 years, best corrected visual acuity in both eyes ≥ 20/25, and stereopsis ≥ 60″arc.

#### 2.1.2. Selection of Adults

Adults older than 21 years of age were included in the study. The ophthalmic examination included monocular visual acuity (Snellen chart at 3 m), biomicroscopy, intraocular pressure measurement, and fundus examination. If any abnormality was found or visual acuity was lower than 20/25, the subject was excluded from the study.

Standard automated perimetry using the Swedish interactive threshold algorithm and the 30-2 program (Humphrey field analyzer; Carl Zeiss Meditec Inc., Dublin, California) was performed in all adults. Subjects were included if visual fields were normal and reliable (fixation loss and false-positive and false-negative responses < 33%).

### 2.2. OCT Scan

The optic disk cube 200 × 200 scan mode measures the ONH parameters and the pRNFL thickness. The ONH stereometric parameters include ONH area, NRR area, average CDR, vertical CDR, and cup volume. The center of the scan circle was manually positioned at the optic disk center in each case for a correct interpretation of the examination. Correct segmentation was verified for each OCT image. Decentered images, with poor signal strength (< 7/10) with eye motion or a blinking effect, were excluded. The system exhibits the average pRNFL thickness and pRNFL thickness of each quadrant (temporal, superior, nasal, and inferior).

The macular cube 512 × 128 scan mode analyzes the ganglion cell (GC) thickness. The software identifies the mGCIPL (from the outer border of the pRNFL to the outer border of the inner plexiform layer [IPL], resulting in a conjugation of the retinal GC layer and the IPL). The mGCIPL thickness analysis is presented with average and minimum values and six sectorial thicknesses (superotemporal, superior, superonasal, inferonasal, inferior, and inferotemporal).

Correct segmentation was checked for each OCT image by co-authors RSC and TN. Manual segmentation is not possible on this device, so whenever segmentation was not correct, the patient was excluded from the study. Good images with Cirrus HD-OCT were defined by signal strength ≥ 7/10 and images that were obtained during visible eye motion or blinking artifacts that were unfocused and those that were poorly centered were excluded. The adult exams were included if all parameters were normal according to the normative database of the software.

### 2.3. Data Analysis

In this study, data were analyzed using the statistical package SPSS for Windows (Version 18.0; SPSS Inc., Chicago, Illinois, United States of America). The continuous variables were compared among groups using a one-way ANOVA test. The chi-square and Kruskal–Wallis tests were used to compare noncontinuous variables between the two groups. *p* values of less than 0.05 were considered statistically significant.

## 3. Results

Adults and children attending the ophthalmology department were contacted to participate in this study. A total of 501 children and 628 adults were evaluated, 172 were selected and agreed to participate in this study, 86 belonged to children, and 86 to adults. There were 41 Children MD, 45 Children, 41 Adults, and 45 Adults NSD.

The characteristics of patients at baseline were balanced between the four groups (Table 1).

**Figure 1:**
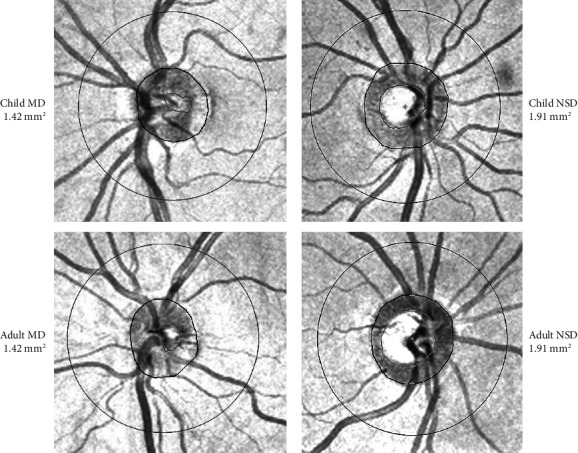
Children with microdisks (MDs) and adults with MD have similar cup sizes and cup–disk ratio, whereas children with normal-sized disks (NSDs) have a smaller cup and a larger neuroretinal rim area than Adults NSD, for the same disk size. Numbers represent the optic nerve head area in mm^2^.

We have compared the groups according to disk size (Table 2 and Figure 1).

Comparing Children and Adults NSD, there was a statistical difference in all ONH parameters. Moreover, there was a difference in the average, temporal, superior, and inferior pRNFL thickness and in the superior sectors of mGCIPL (superior, superonasal, and superotemporal) thickness. On the contrary, the only differences found in the comparison between Children and Adults MD were the NRR area (larger in children, *p* = 0.009) and nasal pRNFL thickness (larger in adults *p* = 0.010).

## 4. Discussion

The main finding of this study is the absence of statistical differences in the cup volume, CDR, and the thickness of pRNFL and mGCIPL between Children and Adults MD, contrarily to NSD. To our knowledge, the absence of a difference in cup size between Children and Adults MD has not been reported before.

Almost a century ago (1923 and 1948), Pickard evaluated the cup size change in a period of 10–15 years, in the same individuals, based on the drawing of the optic disk observed with the direct ophthalmoscope [15, 16]. These studies concluded that there is a continuous enlargement of the cup which goes on after the body has stopped growing. Using OCT measurements, Larsson and El Dairi also found an increase in CDR and cup volume in children with increasing age [17, 18]. In a study comparing children and adults with megalopapilla, we also found that, in adults with megalopapilla, the CDR and cup volume were significantly higher than in children with megalopapilla and the NRR area was thinner [8].

The loss of GCs that occurs through life induces a remodeling of the optic disk head [10, 19]. In MD, the nerve fiber density per disk area is higher than in those with large optic disks, which indicates a crowding of nerve fibers in small ONHs [1]. We believe that in MD, the density of the nerve fibers is so high that the loss of GCs with age does not leave enough space for the fibers to rearrange. This way, the enlargement of the cup that is observed on other disks is not visible in MD. The only parameter that demonstrates the remodeling of the optic disk in MD is the NRR area, which is smaller in adults than in children.

On the contrary, the ONH parameters and the pRNFL thickness of Children and Adults NSD are statistically different, with larger CDR and cup volume and smaller NRR area and lower pRNFL thickness in adults. The only exception is the mGCIPL thickness in NSD that has no significant difference between children and adults. In a previous study, we have demonstrated that the remodeling of the disk is more visible at the optic disk than at the macula because the age-related decline of the pRNFL thickness is higher than the mGCIPL thickness [20].

If the findings in this study are proven to be true, Children MD and Adults MD have a stable small-sized cup. Moreover, the increase of the cup volume in a subject with MD (child or adult) should raise the suspicion of glaucomatous damage, because contrary to NSD and megalopapilla, the physiological enlargement of the cup in MD with age is not to be expected. Also, the normative database of OCT measurements should be adjusted to the disk size and not only for age and sex.

This is a study with a small sample, evaluating subjects who were attending an ophthalmology appointment in a hospital. A bias of selection must be considered because this sample might not be representative of the population. Moreover, ethnicity and axial length were not considered and the variability of the OCT measurements in healthy individuals according to these factors is known [21, 22].

In conclusion, the ONH parameters and pRNFL thickness in NSD are different in children and adults, a consequence of the remodeling of the optic disk with age. On the contrary, MD belonging to children and adults did not have significant differences in ONH parameters and inner retinal layer thickness. Probably the crowding of the nerve fibers observed in MD prevents the enlargement of the cup that occurs in other disks. According to these findings, the evaluation of a glaucomatous lesion in MD should rely on the ONH parameters, and an increase in the cup volume must raise the suspicion of glaucomatous damage, because the physiological enlargement of the cup with age in a MD with age is not to be expected.

## Figures and Tables

**Table 1:** Baseline characteristics of the patient**s**.

	Children MD *n* = 41	Adults MD *n* = 41	*p*	Children NSD *n* = 45	Adults NSD *n* = 45	*p*	All children *n* = 86	All adults *n* = 86	*p*
Age (years) [min, max]	6.55 [4, 11]	52.67 [21, 76]	< 0.001	6.42 [4,10]	55.09 [24, 81]	< 0.001	6.48 [4, 11]	53.95 [21, 81]	< 0.001
Female gender (%)	27 (65.9)	27 (65.9)	0.815	20 (44.4)	20 (44.4)	1.000	47 (54.7)	47 (54.7)	1.000
ONH area (mm^2^) [95% CI]	1.48 [1.47, 1.50]	1.49 [1.47, 1.51]	0.695	1.91 [1.89, 1.93]	1.91 [1.89, 1.93]	0.958	1.71 [1.67, 1.75]	1.71 [1.67, 1.75]	0.741

Abbreviations: MD = microdisk; NSD = normal-sized disk; ONH = optic nerve head.

**Table 2:** Comparison of the optic nerve head's stereometric parameters and inner retinal layer thickness between groups.

	Children MD average [95% CI]	Adults MD average [95% CI]	*p*	d (Effect size)	Children NSD average [95% CI]	Adults NSD average [95% CI]	*p*	d (Effect size)
Neuroretinal rim (NRR) area (mm^2^)	1.36 [1.32, 1.40]	1.50 [1.04, 1.96]	0.008	0.01	1.59 [1.53, 1.65]	1.35 [1.27, 1.43]	< 0.001	0.85
Average CDR	0.27 [0.25, 0.29]	0.35 [0.30, 0.40]	0.221	−0.01	0.39 [0.35, 0.43]	0.51 [0.46, 0.56]	0.001	−0.66
Vertical CDR	0.30 [0.16, 0.44]	0.34 [0.29, 0.39]	0.573	−0.01	0.35 [0.31, 0.39]	0.49 [0.44, 0.54]	< 0.001	−0.82
Cup volume (mm^3^)	0.04 [0.02, 0.06]	0.09 [0.03, 0.14]	0.113	0.00	0.09 [0.06, 0.12]	0.18 [0.14, 0.22]	0.001	−0.64
pRNFL thickness (μm)								
Average	95.17 [92.49, 97.85]	97.07 [94.02, 100.75]	0.295	−0.15	102.58 [99.74, 105.42]	95.47 [93.17, 98.04]	< 0.001	0.86
Inferior	124.85 [119.19, 134.61]	128.20 [123.57, 132.83]	0.352	0.21	134.76 [129.46, 140.06]	124.13 [119.16, 129.10]	0.004	0.65
Superior	122.51 [118.29, 126.73]	120.15 [116.15, 124.15]	0.210	0.09	126.09 [122.11, 130.07]	119.60 [115.75, 123.45]	0.021	0.52
Nasal	68.93 [64.38, 73.03]	77.61 [72.24, 82.98]	0.010	0.21	79.16 [75.04, 83.28]	74.56 [72.85, 77.27]	0.066	0.53
Temporal	65.80 [62.32, 68.48]	62.93 [60.06, 65.17]	0.210	0.17	69.58 [66.51, 72.65]	63.47 [60.92, 66.02]	0.003	0.74
mGCIPL thickness (μm)								
Average	83.98 [82.36, 85.62]	84.66 [82.90, 86.42]	0.323	−0.07	85.87 [84.24, 87.50]	83.53 [81.82, 85.24]	0.051	0.43
Minimum	81.46 [81.30, 81.62]	82.29 [80.54, 84.04]	0.453	−0.09	83.07 [81.34, 84.80]	81.42 [79.69, 83.15]	0.182	0.30
Superior	84.59 [82.63, 86.55]	86.05 [84.97, 88.13]	0.311	−0.13	87.11 [85.03, 89.19]	83.20 [81.27, 85.13]	0.007	0.64
Superonasal	85.61 [83.87, 87.35]	86.80 [84.82, 88.78]	0.355	−0.11	88.27 [86.51, 90.03]	84.91 [83.17, 86.64]	0.008	0.61
Superotemporal	82.29 [80.73, 83.85]	82.88 [81.13, 84.63]	0.624	−0.06	85.31 [83.54, 87.08]	82.78 [81.00, 84.60]	0.049	0.43
Inferior	82.68 [80.89, 84.74]	83.00 [81.12, 84.86]	0.812	−0.03	83.42 [81.50, 85.34]	82.20 [80.36, 84.04]	0.360	0.21
Inferonasal	84.78 [82.14, 85.72]	85.05 [83.17, 86.93]	0.844	−0.02	86.24 [84.54, 88.30]	83.80 [81.89 85.74]	0.054	0.40
Inferotemporal	83.93 [82.26, 85.60]	84.27 [82.60, 85.94]	0.772	−0.03	85.44 [83.80, 87.08]	83.73 [81.99, 85.47]	0.156	0.31

Abbreviations: CDR = cup–disk ratio; mGCIPL = macular ganglion cell–inner plexiform layer; MD = microdisks; NSD = normal-sized disks; pRNFL = peripapillary retinal nerve fiber layer.

## Data Availability

The data that support the findings of this study are available from the corresponding author upon reasonable request.
